# Parasite Communities of Icefish (*Chionodraco hamatus*) in the Ross Sea (Antarctica): Influence of the Host Sex on the Helminth Infracommunity Structure

**DOI:** 10.1371/journal.pone.0088876

**Published:** 2014-02-18

**Authors:** Mario Santoro, Simonetta Mattiucci, Paolo Cipriani, Bruno Bellisario, Francesco Romanelli, Roberta Cimmaruta, Giuseppe Nascetti

**Affiliations:** 1 Department of Public Health and Infectious Diseases, Section of Parasitology, Sapienza University of Rome, Rome, Italy; 2 Department of Ecological and Biological Sciences, Tuscia University, Viterbo, Italy; 3 Department of Experimental Medicine, Sapienza University of Rome, Rome, Italy; University of Minnesota, United States of America

## Abstract

Parasite communities of *Chionodraco hamatus* were investigated from Terra Nova Bay (Ross Sea, Antarctica) during host spawning time. Special attention was given to helminth infracommunities and effect of host sex on its structure. A total of 21 taxa including 5 ecto-parasites and 16 endo-parasites were identified. The number of ecto and endo-parasite species per individual host ranged from 1 to 3 and 3 to 10, respectively, while the mean numbers of parasite specimens per individual host were 4.7 and 1309.7, respectively. The rich abundance of infection suggests a rich concentration of helminth intermediate/paratenic hosts in the coastal waters of Terra Nova Bay. *Chionodraco hamatus* serves as a definitive host for 10 helminth taxa, while it acts as an intermediate/paratenic host for 6 helminth taxa. Larvae of 6 helminth taxa for which *C. hamatus* serves as intermediate/paratenic host represented 98.7% of all specimens found. Of these, the tetraphyllidean and diphyllobothridean cestodes and the nematode *Contracaecum osculatum* s.l. were the most prevalent and abundant. ‘Larval’ infracommunities had significantly higher species richness, total abundance and diversity than ‘adult’ infracommunities, suggesting the important role of *C. hamatus* in supporting the life cycles of those parasites in the study area as a paratenic/intermediate host. Significant differences in the pattern of helminth infracommunities of larval forms between male and female fish were found. These differences could be caused by physiological, and most probably by behavioral differences between sexes suggesting that sex is an important factor influencing parasite burden in *C. hamatus* during reproductive season.

## Introduction

Icefish belonging to Channichthyidae, a family unique among vertebrates in that they lack haemoglobin, live in the cold-stable environment of the Southern Ocean and include between 15 to 17 recognized species [1], [2]. Among the nine species occurring in the Ross Sea (Eastern Antarctica), *C. hamatus* is the most abundant [3]. It has a high-Antarctic type of distribution being limited to shelves close to the Antarctic continent, where it feeds mainly on euphausiid crustaceans and benthic and mesopelagic fishes according to local and seasonal availability [1], [2], [3].

Despite its common occurrence in the Ross Sea, quantitative helminthological studies of *C. hamatus* have been focused so far on specific parasite taxa and limited to occasional examination of incomplete individual hosts [4], [5]. Most studies were on taxonomic features of specific taxa, description of new or re-description of poorly known species [5], [6], larval anisakids identification by genetic markers [7], or pathological changes by larval helminths [8].

In fish, parasite communities may be influenced by both host-related factors (i.e. diet, body size, reproductive behavior, vagility and migratory habits), and habitat-related factors [9], [10], [11]. Because of the difference in body size between sexes, as well as changes during the reproductive season, fish may show different behaviors, vagility and migratory habits, and in turn different diets and/or amount of prey ingested [9], [10], [11], [12], [13]. Since those differences have been described in several icefish species [1], [2], while exposure of trophically transmitted helminths is directly related to the trophic behaviour of fish, we might expect that helminth communities of male and female individual hosts of *C. hamatus* during reproductive season would differ at least in terms of abundance.

Here we report for first time on the parasite community of *C. hamatus* during the spawning season with emphasis on helminths, and we test the hypothesis that males and females show differences in their parasite community structure. In addition, using a parasitological approach we studied the role of this fish species on the trophic food web of Terra Nova Bay (Ross Sea).

## Materials and Methods

### Fish sampling and parasitological identification

This study was approved by the animal ethics committee of “La Sapienza” University of Rome and the Italian Ministry of Education and Research (MIUR). We studied a total of 100 *C. hamatus* sampled in January 2012 (*n* = 50), and February 2013 (*n* = 50) by hand line or net at benthic depths ranging from 110 to 160 m in front of the Mario Zucchelli Station in Terra Nova Bay (74° 41′S – 164° 05′E/74° 41′S – 164° 04′E). Because the fishing activities were not performed in protected areas, no permission was required for this study. Additionally, *C. hamatus* is not an endangered or protected species. Fish were weighed to the nearest 0.1 g and, measured (fork length-FL) to nearest 0.1 cm; the gender was determined before parasitological examination by gonad inspection. *Chionodraco hamatus* is reported to spawn during late summer/autumn [1], [2]. A macroscopic gonad maturity score was recorded to investigate the onset of spawning and sexual maturity (1 = immature; 2 =  resting (mature); 3 =  ripe; 4 =  running ripe; 5 =  spent) [14]. Body condition index (BCI, whole weight/fork length^3^) was calculated as described by Le Cren [15] because it is a good indicator of the general well-being of a fish [16].

Skin, musculature, gills, mouth cavity, visceral cavity, digestive tract, liver, heart, gonads and mesenteries of each fresh individual fish were examined under a dissecting microscope for parasites. For each organ, ecto and endo-parasites were collected, counted, washed in physiological saline, and fixed in 70% ethanol. When encysted, larval helminths were excysted mechanically with the help of a needle. Acanthocephalans, cestodes and digeneans were stained with Mayer's acid carmine and mounted in Canada balsam, whilst nematodes were mounted in lactophenol with cotton blue for identification or frozen to −20°C for genetic identification. Specimens were deposited in the Italian National Antarctic Museum (MNA, Section of Genoa) (accession numbers: from MNA5234 to MNA5254).

A total of 382 larval nematode specimens of *Contracaecum osculatum* s.l. was genetically identified using multilocus allozyme electrophoresis (MAE). Standard horizontal starch gel electrophoresis was performed at those enzyme loci which have proven to be diagnostic between the two sibling species *C. osculatum* sp. D and sp. E and with respect to the other Antarctic species, *C. radiatum* [17], [18]. These are: Malate dehydrogenase (MDH) (EC 1.1.1.37), and Adenylate kinase (EC 2.7.4.3). Details on MAE procedures used for those enzyme-loci analyzed are given in a previous paper [17].

### Data analysis

Apart from analyses based on all parasite taxa, we focused on endo-parasites and on the basis of parasite stage we considered two helminth categories in the description analysis, and interpretation of infracommunity structure (all parasites of different species in the same host individual), i.e. the ‘larval’ infracommunity and the ‘adult’ infracommunity [19]. The ‘larval’ infracommunity was composed of parasite taxa for which *C. hamatus* act as putative intermediate/paratenic host (see Table 1). The ‘adult’ infracommunity included parasite taxa that reproduce in *C. hamatus* (i.e. *C. hamatus* acts as definitive host) (see Table 1). The differentiation between both groups of parasites is obviously justified because of the different ecological role that *C. hamatus* plays in their life cycles. For instance, the lifespan of larvae in intermediate/paratenic hosts is expected to be generally longer than that of adult worms.

**Table 1:** Infection parameters (P: prevalence; Mi: mean intensity), parasite stage “S” (A, adult; L, larva), and known intermediate/paratenic and definitive hosts of the parasite taxa found in 100 *Chionodraco hamatus* from Terra Nova Bay (Ross Sea), Antarctica.

Species	P %	Mi	S	Site in host	Known intermediate/paratenic hosts	Known definitive host
Copepoda						
* Eubrachiella gaini*	28	2.9 (2.2–3.5) [1]–[6]	A	Gill, skin	Absent	Fish
Isopoda						
* Gnathia calva*	9	1.6 (1–2.3) [1]–[3]	L	Gill, skin	Fish	Adult stage in sponges, tunicates and tubes of serpulid worms
Piscicolidae						
* Nototheniobdella sawgeri*	33	4.6 (4.0–5.3) [1]–[8]	A	Gill, skin, mouth	Absent	Fish
* Trulliobdella capitis*	8	2 (1.3–2.6) [1]–[3]	A	Skin	Absent	Fish
* Cryobdella antarctica*	7	2.2 (1.4–3.1) [1]–[4]	A	Gill, skin	Absent	Fish
Nematoda						
* Contracaecum osculatum* s.l.*	100	152.2 (98.9–150.0) [14–1031]	L	Liver, gastric wall, body cavity	Crustaceans?, fish	Weddell seal (*Leptonychotes weddellii*)
* C. radiatum*	86	16.2 (12.8–19.1) [1–57]	L		Crustaceans?, fish	Weddell seal
* Ascarophis nototaenia*	22	3.9 (2.2–5.6) [1]–[18]	A	Stomach	?	Fish
Digenea						
* Derogenes johnstoni*	3	1.6 (1.1–4.5) [1]–[3]			*Derogenes varicus* uses gastropods (*Natica* spp.), crustaceans	Fish
* Elytrophalloides oatesi*	18	4.6 (2.2–5.9) [1]–[15]	A	Stomach	?	Fish
* Genolinea bowersi*	19	1.6 (1.1–2.0) [1]–[4]	A	Stomach	?	Fish
* Gonocerca phycidis*	12	2.0 (1.0–3.0) [1]–[6]	A	Stomach	?	Fish
* Lepidapedon garradi*	3	3 (3.5–9.5) [1]–[6]	A	Intestine	?	Fish
* Neolebouria terranovensis*	43	22.9 (14.9–30.9) [1–86]	A	Intestine	*Neolebouria antarctica* uses crustaceans (*Antarctomysis maxima*)	Fish
*Macvicaria georgiana*	15	5.6 (2.8–8.9) [1]–[18]	A	Intestine	?	Fish
Acanthocephala						
* Corynosoma hamanni*	6	20.6 (4.3–33.9) [8]–[36]	L	Body cavity	Isopods (*Prostebbingia brevicornis*), fish	Weddell seal, leopard seal (*Hydrurga leptonyx*)
*C. pseudohamanni*	5	27.6 (1.0–56.2) [3–52]	L	Body cavity	Isopods (*Cheirimedon femoratus, P. brevicornis*), fish	Seals
* Metacanthocephalus campbelli*	37	8.4 (4.5–12.4) [1–61]	A	Intestine	*M. johnstoni* uses isopods (*C. femoratus*)	Seals
* M. rennicki*	14	3.71 (1.8–5.6) [1]–[12]	A	Intestine	*M. johnstoni* uses isopods (*C. femoratus*)	Seals
Cestoda						
* *Diphyllobothrideans	100	300.8 (261.0–340.5) [67–1061]	L	Liver, gastric wall, body cavity	Crustaceans?, fish	Birds, marine mammals
* *Tetraphyllideans**	100	823.0 (682.8–963.3) [37–3427]	L	Rectum	Crustaceans?, fish	Sharks, skates

Numbers in parentheses represent the 95% confidence interval of each parameter; numbers in square brackets are ranges. Know intermediate/paratenic and definitive hosts in accordance with references detailed in the text [6], [7], [29], [30], [31], [32], [42], [43], [50].

*Contracaecum osculatum s.l. includes the two specie *C. osculatum* D and *C. osculatum* E genetically identified.

**Tetraphyllideans include at least 2 morphological forms.

The Mann-Whitney, Kruskal-Wallis and Chi-squared tests were performed to test, respectively, the influence of size (including FL and weight) and years in the sex of fish and their influence on their infracommunity structure. Mean total abundance, species richness and Brillouin's index of diversity were used as overall descriptors of infracommunities. Mean total abundance is the mean number of individuals of all helminth species, and species richness the number of helminth species harboured by each individual fish. The 95% confidence interval (CI) for prevalence was calculated with Sterne's exact method [20], and for mean values of intensity, abundance, total abundance, species richness and Brillouin's index, with the bias-corrected and accelerated bootstrap method using 20,000 replications [21]. Species richness, mean total abundance and Brillouin's diversity index were compared between ‘larval’ and ‘adult’ infracommunities with the Mann-Whitney U-test for unpaired samples.

A permutation multivariate analysis of variance (PERMANOVA) based on a similarity matrix [22] was used to evaluate whether exist significant differences in the structure of parasite infracommunities (i.e. the number of different parasites taxa living in an infected host) between males and females. A Bray-Curtis similarity matrix was obtained following the fourth-root transformation of the raw intensity data for each taxon [19], and was ‘zero-adjusted’ by adding 1 to all cells [23] due to the high frequency of non-infected individuals (i.e. a zero value in the association matrix). We used the ‘adonis’ function implemented in the package ‘vegan’ of R (R Development Core Team 2011), to partitioning distance matrices among sources of variation. Sex was used to group individuals of *C*. *hamatus*, with the BCI treated as fixed factor. Significance was tested by performing 1,000 permutations of the raw number of individuals of parasites within each group, and a bootstrap pair-wise t-test with 2,000 replications [21] was then used to investigate for differences in parasite assemblage between groups (i.e. males and females).

Finally, to evaluate the contribution to dissimilarity of each individual parasite taxa a SIMPER (Similarity Percentage) analysis was conducted, and multivariate patterns among observations were visualized by means of a non-metric Multidimensional Scaling ordination (nMDS) based on the Bray-Curtis distances [24]. PERMANOVAs were carried out for specific categories of parasites: i) ecto-parasites; ii) endo-parasites (larvae+adults); iii) larval endo-parasites; and iv) adult endo-parasites.

Because parasite body size may be important to understand the structure of parasite communities since, in general, the abundance of a parasite species is related to its body size [25], for the endo-parasite infracommunities we re-ran the PERMANOVA by using the estimated biomass of parasites following George-Nascimento et al. [26], [27]. Briefly, the body mass of each parasite taxon was expressed as the volume (mm^3^) of a cylinder (nematodes and acanthocephalans), an ellipsoid (digeneans), or a cylinder with an ovoid base (tetraphyllideans). For taxa with large bodies and irregular forms (diphyllobothrideans), we measured the volume of displaced water in a beaker. The number of parasites measured for each taxon consisted of at least 20 specimens, then we estimated the whole volume body mass of each taxon within each host species, by multiplying the mean volume body mass of each parasite taxon per the number of the specimens of that taxon in that host.

## Results

### General data

Fish were all spawning individuals with gonad maturity score of 3 or 4. Male individuals were from 242 to 531 g in weight, and from 30 to 39 cm of FL; female individuals were from 267 to 876 g in weight, and from 32 to 40 cm of FL. Mean values ± SD of FL of males (32.493±0.339 cm) and females (35.132±0.356 cm), as well as the mean total weight ± SD (357.831±75.43 g for males and 547.569±131.309 g for females) differed significantly (Mann-Whitney U-test, nFemales  = 55, nMales  = 45, U = 525.5, p<0.001 and U = 223, p<0.0001, respectively). The mean size of fish did not differ between years (Kruskal–Wallis test, P = 0.499). In addition, the number of females and males was independent of year (Chi-squared tests, P>0.8). On the other hand, the factor ‘year’ did not have a significant effect on infection values of any parasite taxa, nor on infracommunity structure (data not shown).

### Parasitological identification and levels of infection

We identified a total of 21 parasite taxa including 5 ecto-parasites (1 copepod, 1 isopod and 3 leeches) and 16 endo-parasites (2 cestodes, 3 nematodes, 4 acanthocephalans, and 7 digeneans) (Table 1). Basic parameters of infection for each parasite taxa plus parasite stage, location in host, and the intermediate/paratenic and final groupings are presented in table 1.

Of the 283 ecto-parasite specimens collected, only those of *Gnathia calva* were all immature forms. Additionally 29% were adult specimens of *Eubrachiella gaini* and 65.7% were adult leech specimens including three species (Table 1).

Of the 130,990 helminth specimens collected, larval forms represented 98.7% of all specimens (85.8% larval cestodes, 12.7% larval nematodes, and 0.2% larval acanthocephalans). Additionally, 0.9% of all specimens were adult digeneans, 0.3% adult acanthocephalans, and 0.06% adult nematodes, all individual forms of these latter three classes were from the gastrointestinal tract (Table 1).

Following the criteria described in Materials and methods, two groups of helminth taxa could be distinguished. First, in 10 species including 1 nematode, 2 acanthocephalan and 7 digeneans, most of the worms were found as adults (Table 1). One copepod and 3 leeches were also found just as adults (Table 1). A second group was composed of 6 helminth taxa (including the four most abundant taxa here found) and one isopod which were found only as larvae (Table 1).

The most abundant taxon, tetraphyllidean cestodes, (mean abundance 823 per host with a maximum value of 3427) represents a mixture of at least two larval forms of morphs including cercoids with monolocular bothridia and accessory suckers and cercoids with bilocular bothridia lacking accessory suckers [28]. Diphyllobothrideans were also very abundant (mean abundance 301 per host with a maximum value of 1061) followed by the nematodes, *C. osculatum* s.l. (mean abundance 152 per host with a maximum value of 1031), and *C. radiatum* (mean abundance 14 per host with a maximum value of 57).

The larval specimens of the *C. osculatum* s.l. included the two cryptic species *C. osculatum* sp. E and *C. osculatum* sp. D. Their identification to the species level by allozymes at those diagnostic loci, allowed to assign 57 larvae (18.2%), among the 382 analyzed, to the sibling species *C. osculatum* sp. E, while 256 (81.7%) were found to correspond to the species named as *C. osculatum* sp. D.

### Parasite communities

The number of ecto-parasite species per individual host ranged from 1 to 3 with a mean number of parasite specimens per individual host of 4.7. The numbers of helminth species per individual host ranged from 3 to 10 with a mean number of worms per individual host of 1309.7. Descriptors of infracommunity structure are shown in table 2. ‘Larval’ infracommunities had significantly higher species richness, total abundance and diversity than ‘adult’ infracommunities (Mann-Whitney U-test, n_larvae_ = 100, n_adults_ = 85, U>20, p<0.001 for all descriptors) but lower dominance (Mann-Whitney U-test, n_larvae_ = 100, n_adults_ = 85, U = 53.7, p<0.001).

**Table 2:** Mean values (95% C.I. in parenthesis and range in square brackets) of 4 parameters of parasite communities calculated for ecto-parasites, endo-parasites (larvae+adults), endo-parasites (only larvae), and endo-parasites (only adults) in 100 *Chionodraco hamatus* from Terra Nova Bay (Ross Sea), Antarctica.

	Ecto-parasites	Endo-parasites (larvae+adults)	Endo-parasites (larvae)	Endo-parasites (adults)
Species richness	1.4 (1.3–3.4) [1.2–1.5]	5.8 (7.2–12.3) [5.5–6.1]	3.9 (4.1–6) [3.8–4]	2.1 (2.4–5.4) [1.9–2.4]
Total abundance	4.7 (0.2–9.1) [4.2–5.2]	1309.7 (65.4–2553.8) [1148.5–1470.7]	1292.7 (64.6–2520.8) [1131.7–1453.7]	19.9 (0.9–38.8) [14.7–25.1]
Brillouin index	0.16 (0.08–0.68) [0.11–0.20]	0.95 (0.93–1.04) [0.90–0.99]	0.89 (0.88–0.97) [0.85–0.92]	0.35 (0.32–0.92) [0.28–0.42]
Berger-Parker index	0.88 (0.42–0.99) [0.85–0.91]	0.60 (0.59–0.65) [0.57–0.62]	0.61 (0.60–0.66) [0.58–0.63]	0.78 (0.45–0.87) [0.74–0.83]

Results from PERMANOVA showed no significant differences in the pattern of ecto-parasite assemblage between males and females of *C*. *hamatus* individuals (F = 0.123, d.f. = 1, p = 0.911). An opposite trend was observed for the helminth infracommunities (larvae+adults), which showed a significant differentiation in the pattern of assemblage, both considering the raw abundance (F = 2.999, d.f. = 1, p = 0.041) and estimated biomasses (F = 3.465, d.f. = 1, p = 0.022). Within these latter infracommunities, adult forms showed no significant differences among sexes regardless of the raw abundance or estimated biomasses (F<1.654, d.f. = 1, p>0.15 in both cases), contrary to what was observed in the larval forms (F>3, d.f. = 1, p<0.04 in both cases). The raw abundances of tetraphyllideans and diphyllobothrideans accounted for most of the variation between sexes in *C*. *hamatus* (SIMPER analysis; 58.97% and 21.05% respectively), followed by *C*. *osculatum* s.l. (13.86%). However, diphyllobotrideans and *C*. *osculatum* s.l. explained much more differentiation when considering the estimated biomasses (SIMPER analysis, 51.9% and 40.19% respectively), with *M*. *campbelli* accounting for only 3.7%.

**Figure 1:**
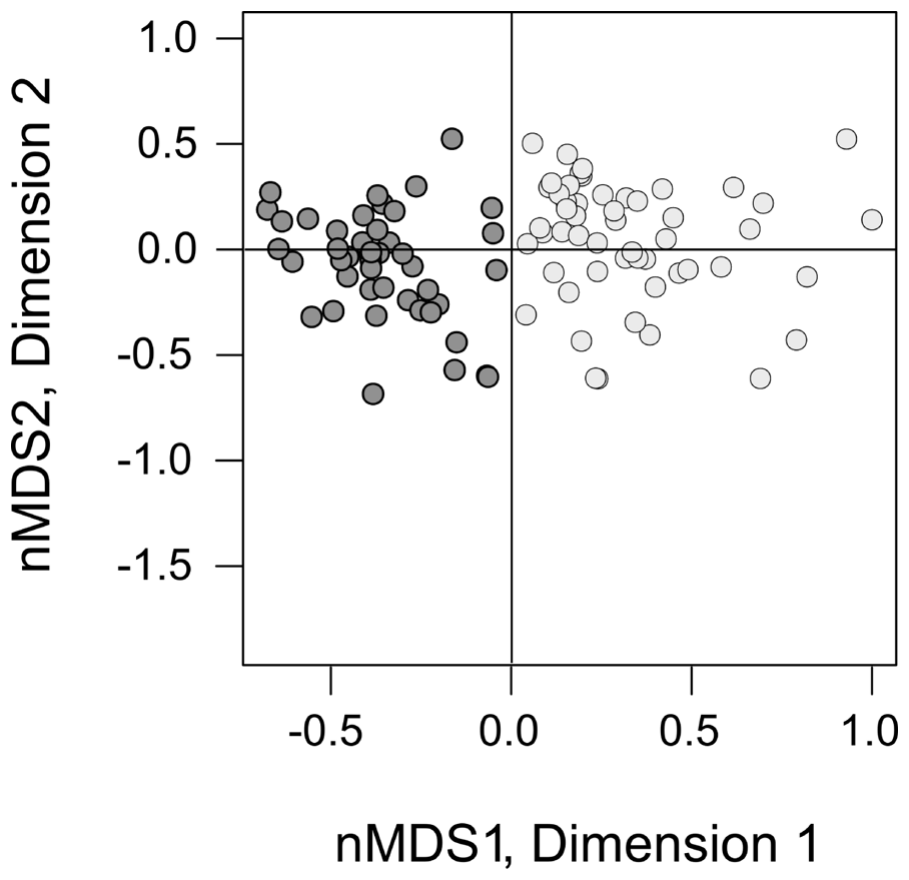
Nonmetric multidimensional scaling (nMDS) ordination plots of parasitic burden by helminths in *Chionodraco hamatus*. Dark gray circles are for male and light gray for female individual fish. Distances between points represent the difference according to the Bray-Curtis dissimilarity.

Therefore, the pattern of assemblage of the parasite infracommunity was mostly related to the larval forms of helminths. In particular, we observed a preferential infestation between sexes of *C*. *hamatus*, with males having on average lower values of BCI than females (Mann-Whitney U-test, n_females_ = 55, n_males_ = 45, U = 581, p<0.001), and this was consistent with the pattern of points separation in the nMDS plot (Fig. 1).

## Discussion

The parasite community of *C. hamatus* from Terra Nova Bay revealed a high parasite diversity with a total of 21 parasite taxa including 5 ecto-parasites and 16 endo-parasites. The total number of endo-parasite taxa goes up to 18 if we consider the two cryptic species (D and E) of *Contracaecum osculatum* s.l. and the two morphs of tetraphyllidean cestodes.

*Chionodraco hamatus* can be considered to act as a definitive host for 10 helminth taxa, 1 copepod and 3 leech species (Table 1). In contrast it serves as an intermediate/paratenic host for 6 helminth taxa, and 1 isopod. This latter group includes the nematodes *C. osculatum* s.l. and *C. radiatum*, and the acanthocephalans *Corynosoma hamanni* and *C. pseudohamanni* whose adults are typical parasites of seals [7], [29]; and diphyllobothrideans and tetraphyllidean cestodes which as adults parasitize birds and marine mammals, and sharks and skates respectively [30], [31]. In addition, *G. calva* which as an adult stage lives in sponges, tunicates and tubes of serpulid worms [32] was found just as a larval form (Table 1). All of parasites here found as adult forms are generalists in Antarctic fish hosts [30].

In general, the most important factors influencing parasite fauna of marine fishes include the feeding habits, the availability of intermediate/paratenic and final hosts, and the host's depth range and migration [9], [11], [33], [34]. The high species richness here found reflects the generalist predatory feeding habits of *C. hamatus* which reaches from shallower waters into the deep-sea (from 0 to 912 m) [1].

The number of helminth taxa here found is similar to that found by Palm et al. [35] in *Chaunocephalus aceratus* from the South Shetland Islands, but consistent differences occurred in taxon composition and infection rates. They found a total of 16 helminth taxa including 2 cestodes, 4 digeneans, 5 nematodes and 8 acanthocephalans, with a mean number of worms per individual host of 7.1 (*versus* 1309.7 here found). However, just 6 of those taxa were in common with our study [35]. Differences in the composition may be related to different environmental features between areas, which in turn influence the presence of intermediate/paratenic hosts. In accordance with Zdzitowiecki [6] and Rocka [31], most parasites of Antarctic fishes show a restricted geographical distribution dependent on the distribution of intermediate hosts within the Antarctic areas. The higher infection levels in individual *C. hamatus* from coastal waters of Terra Nova Bay, as compared to *C. aceratus* from South Shetland Islands, could mainly be related to a ‘dilution effect’ of oceanic conditions and the oligotrophic condition of the pelagic habitat and, consequently, by lower availability of the intermediate hosts necessary to complete the parasites' life cycles in this latter locality [36], [37]. In contrast, the coastal waters of Terra Nova Bay are rich in nutrients, phyto- and zooplankton with a high availability of intermediate/paratenic hosts [3], thus infections of predatory fishes is expected to be higher [36].

‘Larval’ infracommunities had significantly higher community values than ‘adult’ infracommunities suggesting the most important role of *C. hamatus* as intermediate/paratenic host (Table 2). Those included at least three pathogenic taxa for *C. hamatus* [8]. Numerically, larval forms of cestodes including both diphyllobothrideans and tetraphyllideans represented 85.8% of all helminth specimens here collected suggesting an important role of *C. hamatus* in transmitting those cestodes to their definitive hosts. Those cestode larvae are very common in predatory fishes [30]. Their life cycles should involve crustaceans as first intermediate hosts [30]. Unfortunately, matching larvae of diphyllobothrideans with adults has not proved possible so far [31]. Cercoids of tetraphyllideans with monolocular bothridia belong to the genus *Phyllobothrium*, parasitizing adult sharks and skates [28], while cercoids with bilocular bothridia lacking accessory suckers belong to the family Phyllobothriidae or Oncobothriidae [31]. Since sharks are absent from the Ross Sea [38], and skates of *Bathyraja* spp. has been found as the only definitive hosts for tetraphyllidean cestodes to date in eastern Antarctica [30], [31], we speculate that *Bathyraja* spp. are the most important definitive hosts supporting life cycle of those larval forms in Terra Nova Bay. On the other end, from the biological point of view it suggest *C. hamatus* as important prey of *Bathyraja* spp.

The larval stages of *C. osculatum* s.l. represented the third most abundant taxon. Crustaceans are considered as first intermediate host and fishes as intermediate/paratenic hosts while the only definitive host identified so far for both Antarctic anisakid species is the Weddell seal (*Leptonychotes weddellii*). Siegel [39] observed that both *C. aceratus* and *C. hamatus* are virtually free of larvae of *Contracaecum* spp. before becoming demersal. He noted in *C. aceratus* a dramatic increase of larvae from the infection-free planktotrophic stages at <20 cm length to 80/100% infestation in the demersal piscivorous >30 cm length stages, suggesting that intermediate hosts of *Contracaecum* spp. occur in demersal environments. In addition, since none of 2013 individuals of *C. gunnari* (a feeding specialist on krill) were infected with *Contracaecum* spp., Siegel [39] concluded that krill is not an intermediate host for these anisakid nematodes. The higher relative proportion of *C. osculatum* sp. D respect to E here found in *C. hamatus* suggests that prey which harbour the infective stages of spp. D and E are different, and differently ingested by the fish.

The most representative group of helminths found in the present study as adults was that of Digenea with 7 species followed by Acanthocephala with just two species (*Metacanthocephalus* spp.). Life cycles of Antarctic helminths here found are poorly known. Zdzitowiecki [6] suggested that all digeneans maturing in Antarctic bony fishes use mollusks as the first intermediate hosts and invertebrates as second intermediate hosts. To date, only metacercariae of *Neolebouria antarctica* have been found in the Antarctic crustacean (*Antarctomysis maxima*) [40]. According to Zdzitowiecki [29] acanthocephalans maturing in Antarctic fishes (e.g. *Metacanthocephalus* spp.) have two hosts in their life cycles, whereas species maturing in Antarctic birds and pinnipeds (e.g. some *Corynosoma* spp.) have in addition a third paratenic host, a teleost. Amphipods are recorded as the intermediate hosts of *Corynosoma* spp. and *Metacanthocephalus* spp. in Antarctic waters [41], [42], [43].

Although host sex has been listed as a factor that may influence the parasite burden of individuals, statistical inequalities between sexes are uncommon in fishes and may depend on morphological, physiological, and behavioral differences which may vary with the host-parasite system studied [12], [44]. It might be plausible to think that the larger size of females would be related to their heavier parasite burden since a large host would have more space, more flux of energy (i.e. food), and more microhabitats for parasites than a small host. However, it has been suggested that parasite numerical density would decrease as host body weight increases because a large host has lower specific metabolic rate (i.e., flux of energy per gram) so there would be a smaller number of parasites per gram of host [27]. Recently, Poulin and George-Nascimento [45] found that maximum parasite biomass per gram of host is independent of host mass (i.e. larger fish hosts can support the same parasite biomass per gram as small hosts).

Higher prevalence and intensity of infection of parasites in females have been related to investment in reproduction which is more costly than that in males, making females more susceptible to parasite infection in periods of investment in gonad development [12], [46]. On the other hand high testosterone levels may cause immunosupression in males during the reproductive season, making them more susceptible to parasite infection than females [12], [47], [48].

Because physiological and hormonal changes occur in both sexes during reproductive season, a plausible explanation for heavier parasite burdens in females could be related to different behaviours among sexes. Females may ingest higher amount of different groups of crustaceans which represent the first intermediate hosts of most abundant larval taxa here found. It has been reported that males of icefish move inshore for spawning about one month earlier than females where they establish territories prior to spawning [1], [49]. Kock [1] reported that at least three icefish species (*Chaenodraco wilsoni*, *Pagetopsis macropterus* and *C. aceratus*) deposit their eggs on the seafloor, where they are guarded tenaciously by the males [48]. A considerable amount of energy is invested in establishing territories and guarding strategy. This limits foraging time strongly and, in turn, reduces male body condition [1], [49]. By reducing the ingestion of intermediate hosts, males in turn may decrease ingestion of parasites and show lower values of infection than females. This could also explain why males have on average lower values of BCI than females.
